# Progress of immune checkpoint inhibitors therapy for non-small cell lung cancer with liver metastases

**DOI:** 10.1038/s41416-023-02482-w

**Published:** 2023-11-09

**Authors:** Fan-jie Qu, Yi Zhou, Shuang Wu

**Affiliations:** Department of Oncology, Affiliated Dalian Third People’s Hospital of Dalian Medical University, 116033 Dalian, China

**Keywords:** Non-small-cell lung cancer, Cancer immunotherapy

## Abstract

Nearly one-fifth of patients with non-small cell Lung Cancer (NSCLC) will develop liver metastases (LMs), and the overall treatment strategy of LMs will directly affect the survival of patients. However, some retrospective studies have found that patients receiving chemotherapy or targeted therapy have a poorer prognosis once LMs develop. In recent years, multiple randomised controlled trials (RCTS) have shown significant improvements in outcomes for patients with advanced lung cancer following the introduction of immune checkpoint inhibitors (ICIs) compared to conventional chemotherapy. ICIs is safe and effective in patients with LMs, although patients with LMs are mostly underrepresented in randomised clinical trials. However, NSCLC patients with LMs have a significantly worse prognosis than those without LMs when treated with ICIs, and the mechanism by which LMs induce systemic anti-tumour immunity reduction is unknown, so the management of LMs in patients with NSCLC is a clinical challenge that requires more optimised therapies to achieve effective disease control. In this review, we summarised the mechanism of ICIs in the treatment of LMs, the clinical research and treatment progress of ICIs and their combination with other therapies in patients with LMs from NSCLC.

## Background

Nearly one-fifth of patients with non-small cell Lung Cancer (NSCLC) will develop liver metastases (LMs), which have the worst prognosis among lung cancer single organ metastases, with a median survival of only 4 months [1–3]. In the course of clinical diagnosis and treatment, once LMs occurs, it is often accompanied by metastasis of other sites [1]. A study involving more than 20,000 lung cancer patients yielded three months of median overall survival (OS) in patients with LMs [2], and patients with LMs had a 53% higher risk of death than those with central nervous system metastases. The more liver metastases, the worse the survival rate [3]. However, LMs of lung cancer have not received the attention it deserves compared to brain metastases. There are relatively few clinical trials and literatures on LMs of lung cancer. Most lung cancer patients with LMs do not respond well to chemotherapy, although chemotherapy has long been the standard of care for metastatic lung cancer [4–7]. For example, a retrospective analysis published in 2000 showed that NSCLC patients with LMs who received first-line chemotherapy had a significantly increased risk of death compared with patients without LMs [4]. The median overall survival of patients with NSCLC liver metastases receiving standard chemotherapy is not more than 10 months [5–7]. Therefore, holistic treatment strategies for lung cancer LMs will directly affect patient survival, and traditional chemotherapy regimens show small clinical benefits. Although the development of targeted therapy has improved the prognosis of metastatic lung cancer. However, targeted therapies can only cover patients with driver mutations, and the results of these studies on targeted therapy also generally indicate a poor prognosis for patients with LMs [8, 9]. Therefore, there is an unmet clinical need for the treatment of lung cancer LMs.

Immunotherapy has transformed the treatment of many cancers with great success. In recent years, a number of randomised controlled trials (RCTS) have shown that ICIs can significantly increase the rate of OS in patients with advanced lung cancer compared to conventional chemotherapy [10–13]. For patients with advanced NSCLC, ICIs such as programmed cell death-1 (PD-1)/ programmed cell death ligand-1 (PD-L1) inhibitors have been approved for first-line treatment [14]. However, the effect of LMs on the efficacy of ICIs remains inconsistent and controversial, so there is no consensus on the best treatment plan for patients with NSCLC liver metastasis.

Some studies have identified LMs as an independent poor prognostic factor in patients who received ICIs [3, 15, 16]. In a retrospective study receiving pembrolizumab for NSCLC, Tumeh et al. discovered that NSCLC patients with LMs had a significantly shorter progression-free survival (PFS) (1.8 vs. 4.0 months, *p* = 0.0094) and lower objective response rate (ORR) (28.6 vs. 56.7%) than patients with non-liver metastasis, and they confirmed that LMs was predictive of reduced response and poor outcome independently [15]. Kitadai et al. [3] and his team performed ICIs therapy including nivolumab, pembrolizumab, and atezolizumab in 215 patients with advanced NSCLC, 41 of whom developed LMs. The results showed that the ORR of patients with LMs was only 22.5%, and the PFS and OS were shorter. A meta-analysis [16] involving 12 RCTS showed that NSCLC patients with LMs obtained less PFS and OS benefit from ICIs treatment compared with patients without LMs, suggesting that liver metastasis could be considered an independent prognostic risk factor. But there are less consistent conclusions. A retrospective study [17] from Japan found the clinical characteristics of NSCLC patients with LMs treated with nivolumab, with younger age, worse ECOG score, and more metastatic sites in patients, compared with patients without LMs. The researcher found that these baseline clinical characteristics was associated with shorter PFS times, not LMs.

However, in patients with NSCLC with liver metastases, ICIs therapy still has a survival advantage over conventional therapy [6, 16, 18–21]. Pooled analyses of CheckMate017 and CheckMate057 showed that patients with LMs could benefit from second-line nivolumab over conventional docetaxel chemotherapy [6]. The final analysis of KEYNOTE-189 in 2021 showed that patients with LMs could benefit from first-line pembrolizumab plus chemotherapy, although the benefit was lower than that of patients without LMs [18]. Another retrospective study suggested that ICIs combined with cytotoxic agents may be more effective than ICIs alone in NSCLC with liver metastasis [19]. Another Real-World Study came to a similar conclusion [20], among 648 patients with advanced NSCLC treated with ICIs, 61 of whom developed LMs. The results showed that patients with LMs receiving PD-1/PD-L1 inhibitors were effective (ORR: 29.5%, disease control rate: 72.1%, PFS: 6.4 months, OS: 15.2 months). The response rates still exceeded those reported for other therapies, although the efficacy was worse than those without LMs. That meta-analysis of 12 RCTS [16] also showed that ICIs therapy (ICIs monotherapy, ICIs combined therapy) had a survival advantage in patients with LMs compared to standard therapy (PFS HR, 0.77; 95%CI, 0.61–0.97; OS HR, 0.78; 95%CI, 0.68–0.90). In a real-world retrospective study [21] of 1470 advanced NSCLC patients, 234 (15.9%) developed LMs at initial diagnosis. Studies showed that patients with LMs were less likely to respond to cytotoxic drugs or targeted therapies. However, patients treated with ICIs had significantly longer OS than those treated with chemotherapy (11.7 vs. 4.4 months, *P* < 0.001), and there was no significant difference in OS with or without LMs (11.7 vs. 13.0 months, *P* = 0.968), suggesting that LMs is possible not an independent prognostic factor for ICIS-treated NSCLC patients.

To sum up, although the presence of LMs is associated with poor prognosis, clinical studies have shown that ICIs can provide survival benefits compared with previous therapies, especially in some combination therapies for lung cancer LMs, and maybe a new opportunity to treat patients with lung cancer LMs. Therefore, it is necessary to analyse and explore the effect and future development direction of immunotherapy in patients with LMs of NSCLC. To this end, we reviewed the immune microenvironment of lung cancer LMs and the immune mechanism and clinical research evidence of ICIs therapy, hoping to raise the attention of lung cancer LMs and seek better immunotherapy strategies through our work.

## Tumour microenvironment and Immunotherapy Mechanisms of ICIs for LMs from NSCLC

ICIs promotes immune response by blocking immune system regulatory checkpoints and inhibiting tumour growth. The mechanism is that the blockade of immune checkpoints leads to reactivation of the immune response of T cells to tumour cells [22, 23]. However, for immunotherapy, the liver may be organ-specific.

Studies suggest that the liver has immunomodulatory function and can maintain local and systemic immune tolerance to both autoantigens and foreign antigens [24–28]. The mechanism of immune tolerance is mainly attributed to the abundant immune active cells in liver, including liver sinusoidal endothelial cells (LSEC) and Kupffer cells (KC), hepatic stellate cells (HSC), and dendritic cells (DC), which play the roles of antigen presentation, immune regulation, and immune tolerance [24–27]. They can also induce the differentiation of circulating immune cells into regulatory immune cells, such as regulatory T cells (Tregs). Under the cooperation of resident cells and circulating cells, a complex regulatory system is formed to maintain liver immune tolerance [28].

Limmer et al. reported that LSEC could cross-presenting soluble exogenous antigen to CD8+ T cells and the outcome is CD8+ T cell tolerance which thereby induce protective immunity against infecting microorganisms [24]. KC have been found a self-sustaining, liver-resident population of macrophages and promotes the restoration of tissue integrity following liver injury or infection [25]. Chen et al. demonstrate that HSC inhibit the entire acquired immune system by inducing the expansion of bone marrow-derived inhibitory cells (MDSC) [26]. These large numbers of innate and adaptive immune cells in the liver constitute a complex immune microenvironment that together participate in the immune regulation of the liver, not only for the detection, capture and removal of pathogens from the blood, but also to ensure that inappropriate immune responses do not target non-pathogenic exogenous blood-borne molecules. It is this balance between activation and tolerance that characterises the liver as a specific immune organ [27]. Based on this balance, although the liver is constantly exposed to bacterial components and dietary antigens flowing from the gastrointestinal tract via the portal vein due to its unique blood supply, the liver can maintain a certain level of immune tolerance to balance the clearance of bacterial pathogens while avoiding excessive inflammation caused by the non-pathogenic intestinal environment [28].

Studies in the fields of transplantation immunity have also proved that liver is an organ prone to inducing immune tolerance. In the research field of liver transplantation, it has been found that different from heart or kidney allografts, liver allografts can be spontaneously accepted in mice, rats, pigs and even humans, showing good histocompatibility, and even without the use of immunosuppression in some cases [29, 30]. In addition, liver allotransplantation enables the recipient to develop tolerance to other transplanted organs from the same donor, suggesting that the liver can transmit this immune tolerance effect to other organs, thereby inducing systemic immune tolerance [30, 31].

But it is not clear whether liver immune tolerance mechanisms contribute to cancer outcomes. It is also unclear whether the reduction of systemic anti-tumour immune response can be induced after liver metastasis. Some studies have found that tumour microenvironment (TME) phenotype is a key factor in determining the effect of immunotherapy, and the specificity of TME in different organs may be an important reason for the significant difference in the response of patients harbouring different organ-specific metastases to immunotherapy [32, 33]. Osorio’s team [34] confirmed that different organ sites had different responses to ICIs, and lymph node and liver metastasis were the most responsive organs and the least responsive organs, respectively. Among 761 baseline lesions (58 LMs) in NSCLC patients treated with PD-1 inhibitor monotherapy, LMs showed the worst response. Previous relevant studies have shown that lymph nodes were among the most responsive, which may be explained by the local populations of T cells and the role of tumour-draining lymph nodes in priming and conditioning antigen-specific responses to PD-1 blockade [35, 36]. But LMs had among the poorest responses to PD-1 blockade, which may be explained by the liver appears to have distinct immune tolerance affecting local [37] and systemic [27, 38] immunity.

Some translational studies have found that the presence of tumour antigens in the liver leads to systemic suppression of anti-tumour immunity and may lead to resistance to immunotherapy [39, 40]. Yu et al. found in multiple mouse models that tumour antigenic specific CD8+ T cells could be “syphoned” into the liver, leading to systemic “immune desert”. Similarly, they found that the number of peripheral blood T cells in patients with liver metastasis decreased, and the diversity and function of tumour T cells decreased, which ultimately reduced the effect of immunotherapy [39]. Tumeh et al. found in biopsy samples that CD8+ T cell density at the edge of invasive tumours in patients with liver metastasis was lower than that in patients without liver metastasis (liver metastasis + group, *n* = 547 ± 164.8; Liver metastases–group, *n* = 1441 ± 250.7; P & lt; 0.016), and this was associated with decreased ICI response rate and shortened PFS in patients with liver metastases with NSCLC [15]. Another study also found phenotypic changes of CD8+ tumour-infiltrating lymphocytes (TILs) in distal biopsy site of patients with liver metastasis, supporting the possibility that invasion of cancer cells into the liver may trigger liver-specific tolerance mechanisms, thereby reducing the efficacy of systemic antitumor immunotherapy [38]. Other studies have found that CD8+ T cell infiltration in liver lesions is significantly less than in non-liver lesions, suggesting that liver metastasis is related to CD8+ T cells and may influence treatment outcomes through liver-induced peripheral tolerance [3, 20].

In addition, using a dual-tumour immunocompetent mouse model, Lee et al. found that two groups of cells were significantly increased in the liver tumour group, including CD11b + Ly6G + Ly6C - granulocytes and monocytic myeloid-derived suppressor cells (M-MDSCs), both of which have the ability to significantly inhibit immune cell response [40]. Some studies have found that in hepatocellular carcinoma, MDSC is a group of immature myeloid-derived suppressor cells derived from bone marrow cells, have strong immunosuppressive activity and influence antigen presentation [41]. Millrud et al. believed that MDSC has significant diversity and plasticity, and can mainly play an immunosuppressive role in the liver immune microenvironment by differentiating into macrophages, neutrophils and dendritic cells (DC) in different environments [42].

The researchers also found that liver-specific cell components such as KC and HSC play different roles in different stages of liver metastasis. In the early stage of metastasis initiation, KC can clear and kill circulating metastatic cells and play an anti-tumour role, while after adhesion to tumour cells, KC plays a pro-tumour role [25]. Meanwhile, HSC indirectly inhibit the entire acquired immune system by inducing the expansion of bone MDSC [26].

These findings suggest that liver-specific cellular components and tumour-related immune cells mostly play an immunosuppressive role in the liver immune microenvironment, which may be the reason for the poor immunotherapy effect in the population with NSCLC liver metastasis. With the in-depth understanding of the TME phenotype after liver metastasis and the exploration of the formation mechanism of systemic immunosuppression in liver metastasis, it will help guide us to seek better clinical immunotherapy strategies. Based on the current study, most investigators have explored comprehensive therapies to address liver immunosuppression and improve the efficacy of immunotherapy [39, 40]. Lee et al. found that systemic immunosuppression is related to the up-regulation of intratumoral CD11b+ monocytes and the synergistic activation of these cells and Tregs. Anti-PD-1 monotherapy cannot reverse the dysfunctional immune state, and the combination of Treg and CD11b+ monocyte targeting drugs may be required [40]. Yu et al. found in preclinical models that liver-directed radiotherapy eliminated immunosuppressed liver macrophages and reduced liver syphoning of T cells, thereby increasing peripheral blood T cell survival. Therefore, the combination of liver-directed radiotherapy and immunotherapy can promote systemic anti-tumour immunity by reshaping the liver immune microenvironment [39].

In summary, the liver is an organ that is easy to induce immune tolerance, and the tumour microenvironment of liver metastasis and the mechanism of inducing systemic immunosuppression are extremely complex. We know that LMs remain an important unmet clinical challenge in immuno-oncology, and it is of great clinical significance to study how to overcome the immune tolerance of LMs and determine effective immunotherapy strategies.

## ICIs monotherapy

Systemic therapy remains the primary treatment after LMs. There is increasing evidence that immunotherapy alone can benefit these patients and can be tolerated by them, despite the poor prognosis of liver metastases in NSCLC [6, 15, 43, 44]. However, these ICIs data for LMs are mainly from subgroup analyses of clinical trials and retrospective cohort studies.

CheckMate057 [10] and CheckMate017 [11] were the key studies that allowed Nivolumab to obtain FDA second-line indications for lung squamous cell carcinoma and non-squamous NSCLC, respectively. Vokes et al. [6] report the results of 3-year follow-up of the CheckMate017 and CheckMate057 studies, including subgroup analyses of LMs patients. Of the 854 randomised patients, 193 were determined to have LMs after baseline examination. In patients with liver metastasis, the OS in the Nivolumab treatment group was 6.8 months, which was only 1 month longer than that in the chemotherapy group and lower than the average of the general population (11.1 months). Although patients without liver metastasis benefited more from immunotherapy, Nivolumab also significantly prolonged OS in patients with liver metastasis than docetaxel, and the hazard ratio was consistent with results in the overall study population [6]. After longer follow-up, nivolumab continued to show superior OS, PFS, and DOR benefits over docetaxel in multiple subgroups, including LMs, although patients with LMs had poorer outcomes than those without LMs [43]. Compared with chemotherapy, Nivolumab was generally well tolerated and no new safety concerns were identified [6, 43]. The incidence of treatment-related liver adverse events (mainly grade 1–2 liver enzyme elevation) was slightly higher (10%) in patients with liver metastasis treated with nivolumab [6].

Tumeh et al. [15] analysed 165 NSCLC patients with treated with pembrolizumab, and the results showed that 46 patients with liver metastasis had worse prognosis than 119 patients without liver metastasis and the median PFS in patients with or without liver metastasis were 1.82 months and 4.03 months, respectively. Although the response rate in the liver metastasis subgroup was low, pembrolizumab still exceeded that of other previous therapies.

In the pooled analysis of Study 1108 and ATLANTIC [44], the efficacy and safety of 143 patients with LMs treated with durvalumab were observed. In both studies, the median overall survival (OS) was shorter in patients with LMs than in patients without LMs. An interesting finding from both studies was that patients with PD-L1 expression ≥25% in TCs were associated with a better OS prognosis (study 1108, adjusted HR, 0.63, *P* < 0.01; Atlantic study, adjusted HR, 0.60, *P* < 0.01; respectively), independent of liver metastasis. It is suggested that patients with liver metastasis with high expression of PD-L1 may also benefit from ICIs. However, liver injury was observed in 19% of durvalumab-treated patients and is associated with a greater likelihood of tumour progression and death during follow-up. Using multivariate regression analysis, the development of liver injury during treatment as well as baseline hepatic metastases were independently associated with mortality during follow-up [45].

Combination of the above, ICIs has great potential for the treatment of LMs from lung cancer, is expected to be as systemic treatment except chemotherapy and targeted therapy which used to control the LMs lesions; and the preliminary evidence of many research suggests that immunotherapy will not increase the additional risk in patients with LMs from NSCLC. Unfortunately, the effective rate of ICIs monotherapy is low, and most of the evidence from these studies is retrospective analysis and should be interpreted with caution.

## ICIs combination therapy

### ICIs combined with chemotherapy

Immunotherapy holds the potential to induce durable responses, but only a minority of patients currently respond to ICIs monotherapy. The main function of cytotoxic drugs is to reduce the tumour burden by directly killing tumour cells. In the process of destroying cancer cells, the tumour releases tumour-related antigens, which can enhance the immune system’s ability to recognise tumour cells, while reduce the immunosuppressive tumour microenvironment [46, 47]. Preclinical studies have also found that chemotherapy agents deplete immunosuppressive cells, such as Tregs and MDSCs, and thus promote the development of local immunity to positive equilibrium [48, 49]. For this reason, such a mechanism may be particularly appropriate for liver lesions that are heavily affected by Tregs. In addition, immunotherapy can reverse the chemotherapy resistance of tumour cells, thereby improving the sensitivity of tumour cells to chemotherapy drugs and reducing the toxic effects of chemotherapy drugs [50]. Therefore, ICIs combined with chemotherapy has a synergistic killing effect on tumour cells, and chemotherapy is an ideal partner in combination with immunotherapy.

KEYNOTE-189 was a randomised, double-blind phase III study comparing the efficacy of chemotherapy combined with pembrolizumab or placebo in patients with metastatic NSCLC [5]. In a subgroup analysis of 115 patients with liver metastasis, pembrolizumab combined with chemotherapy significantly prolonged the median OS (12.6 months vs. 6.6 months, HR = 0.62, 95%CI: 0.39~0.98; *P* < 0.001). The HR of OS in patients with liver metastasis and those without liver metastasis was similar [18]. A pooled analysis was reported in 2020, including three studies, KEYNOTE-021 cohort G, KEYNOTE-189, and KEYNOTE-407 [51]. The study showed that first-line chemotherapy combined with pembrolizumab extended survival in patients with liver metastasis, although the benefit was relatively lower than in patients without liver metastasis. The results of IMpower131 subgroup analysis showed that Atezolizumab combined with chemotherapy achieved a trend of benefit in PFS in the liver metastasis subgroup compared with carboplatin combined with albumin paclitaxel alone (5.5 months vs. 4.2 months, HR = 0.77, 95% CI, 0.54~1.10) [52].

In a meta-analysis that included 8 RCTs [53], the effect of LMs on the efficacy of PD-1/PD-L1 inhibitors combined with chemotherapy as first-line treatment in lung cancer was evaluated. In patients with LMs, compared with chemotherapy alone, PD-1/PD-L1 inhibitor plus chemotherapy could decrease the risk of progression by 31% and risk of death by 21% (HR = 0.69;95%CI,0.58–0.81; and HR = 0.79; 95%CI,0.62–0.80, respectively), suggesting that lung cancer patients with and without LMs could obtain comparable efficacy.

There are also inconsistent conclusions. In IMpower130 [54] and IMpower132 [55] studies, no significant OS benefit was shown in the subgroup of patients with liver metastasis receiving ICIs in combination with chemotherapy. Some scholars believe that cytotoxic drugs can also damage the anti-tumour function of immune cells, thus reducing the efficacy of ICIs. For example, Anestakis et al. reported that carboplatin treatment can increase the induction of bone marine-derived suppressor cells and deplete CD8+T cells, which may inhibit cellular immunity [56].

In addition, in a recent multicenter retrospective study, patients receiving pembrolizumab first-line therapy (with or without chemotherapy) in the LMs subgroup had even longer PFS in the monotherapy group than in the combination group (*p* = 0.048, HR 0.41, 95% CI 0.16–1.02). However, the researchers later found that this may be because the monotherapy group contained more patients with high PD-L1 expression [57].

To sum up, most scholars believe that although cytotoxic drugs and ICIs alone have relatively low efficacy in NSCLC with liver metastasis, their combination may have synergistic effects. Therefore, patients with LMs may benefit more from ICIs therapy combined with chemotherapy than chemotherapy or immunotherapy alone.

### Anti-CTLA-4 combined with anti-PD-1 therapy

Preclinical evidence shows that combination immunotherapy may overcome the acquired resistance of ICIs monotherapy, through the increase in T-cell infiltration and the reduction of regulatory T-cells, which could enhance the effect of ICIs [23, 56]. Cytotoxic T lymphocyte antigen-4(CTLA-4) mainly inhibits T cell activation during the initiation phase [58]. Considering the immune tolerance of the liver, the combination of CTLA-4 and PD-1 blockers is expected to have a synergistic effect to achieve better outcomes in patients with NSCLC liver metastasis. In addition, studies suggest that CTLA-4 monoclonal antibody is independent of PD-1/PD-L1 pathway, and therefore has certain efficacy even in PD-L1 negative populations [59, 60].

The CheckMate 277 study [59] evaluated the efficacy of Nivolumab in combination with ipilimumab or chemotherapy in patients with advanced or metastatic NSCLC and showed that the combination significantly improved outcomes compared with chemotherapy (2-year ORR: 40.1% vs. 29.7%, *P* < 0.05; Median OS: 17.1 months vs 13.9 months, *P* = 0.007). Overall survival benefited from Nivolumab plus Ipilimumab in most subgroups, including those with bone and central nervous system metastases. Unfortunately, LMs group did not (median OS: 9.5 months vs 11.9 months, HR = 1.5, 95%CI, 0.74~1.49) benefited from double immunity. At the same time, the combination regimen also caused relatively more adverse reactions than monotherapy (grade 3 or 4 adverse events rate: 32.8% vs 19.4%), suggesting the treatment strategy needs to be further optimised according to the patient situation [59].

The CheckMate 9LA trial evaluated the efficacy and safety of dual immunotherapy with nivolumab and ipilimumab combined with 2 cycles of chemotherapy in patients with advanced or metastatic NSCLC. Results showed survival benefits in the experimental group compared with the control group in the overall population and in most subgroups regardless of the expression of PD-L1, while no new safety signals were observed [61, 62].

Although most current studies have not disclosed data for the liver metastasis subgroup, overall, the data from these prospective clinical trials demonstrate the safety and efficacy of anti-CTLA-4 + anti-PD-1 in the treatment of metastatic NSCLC, as well as support its use in patients with liver metastasis. However, attention should be paid to adverse drug reactions and more prospective clinical studies are expected to explore them in the future.

### ICIs combined with antiangiogenic drugs

Preclinical studies have shown that a variety of cellular components in the immune microenvironment, such as tumour-associated macrophage (TAM), MDSC and tumour-associated neutrophils (TAN), play an important role in promoting angiogenesis [63]. Hepato-specific KC and HSC can secrete vascular endothelial growth factor (VEGF) and other proangiogenic factors to promote angiogenesis. On the one hand, activation of these angiogenesis-related pathways can promote the formation of immunosuppressive microenvironments. On the other hand, VEGF and angiopoietin-2 (Ang2) can reduce expression of endothelial cell surface immune checkpoint inhibitor receptors [e.g., PD-1, CTLA-4, lymphocyte-activation gene 3 (LAG3) and T cell immunoglobulin and mucin domain-containing protein 3 (TIM3)] to recruit more innate immune cells (such as TAM and MDSC), thereby influencing the infiltration of cytotoxic T lymphocytes in the immune microenvironment [64].

Further studies revealed that VEGF was overexpressed in liver metastasis and had a negative immunoregulatory effect, while the addition of bevacizumab can improve the immunosuppressive microenvironment by blocking VEGFR, thus promoting anti-tumour effects [65, 66]. Studies have found that antiangiogenic drugs have immunomodulatory effects, including antigen presentation and T cell activation, and even increase T cell infiltration in tumours after inhibiting VEGF [67]. In NSCLC murine models, immunotherapy combined with antiangiogenic therapy produced higher level of tumour-infiltration lymphocytes compared with other treatments [68]. There is increasing evidence that anti-angiogenic therapy has synergistic effects in combination with immunotherapy[67, 68]. Based on these mechanisms, combined antiangiogenic therapy may be an improved measure to improve the efficacy of immunotherapy for liver metastasis in NSCLC.

First of all, the role of antiangiogenic therapy in patients with NSCLC liver metastases is worthy of affirmation. The antiangiogenic agent Bevacizumab has been approved for the treatment of NSCLC based on the results of the Phase III study of ECOG4599 [69]. Subgroup analysis of ECOG4599 showed that bevacizumab combined with chemotherapy was found to prolong survival in the LMs subgroup and that people with liver metastasis benefited more from bevacizumab than from other drugs [69]. A real-world study came to the same conclusion [70].

In recent years, the researchers explore the potential of combining immunotherapy with antiangiogenic treatment option for patients with advanced NSCLC. Some clinical trials have shown that the combination of ICIs therapy and bevacizumab is effective for NSCLC patients with liver metastasis and the trial of antiangiogenic drugs combined with immunotherapy has entered the phase III clinical stage.IMpower150 [71] showed that Atezolizumab combined with bevacizumab, carboplatin, and paclitaxel (ABCP group) had a significant OS and PFS benefit compared to bevacizumab combined with carboplatin combined with paclitaxel (BCP group) in naive patients with non-squamous non-small-cell lung cancer [71]. Reck et al. reported the efficacy of ABCP group versus BCP group in LMs subgroups. Compared with BCP, ABCP led to significantly improved overall survival (OS:13.3 months vs. 9.4 months, HR = 0.52,95% CI: 0.33–0.82) and progression-free survival (PFS:8.2 months vs 5.4 months, HR = 0.41,95% CI: 0.26~0.62) [71]. The final exploratory analysis of the long-term follow-up IMpower150 study showed that OS in the ABCP group was still significantly better than that in the BCP group in the liver metastasis subgroup (HR = 0.68; 95% CI: 0.45–1.02) [7], suggesting that immunotherapy combined with antiangiogenic drugs is a potential treatment option for patients with advanced NSCLC liver metastases. However, it is worrisome that the combination regimen is associated with a higher incidence of treatment-related adverse events. The study showed that 223 patients (57%) in the ABCP group had grade 3-4 treatment-related events [71].

Advanced hepatocellular carcinoma and advanced NSCLC liver metastases have similarities in the immune microenvironment of the liver. In fact, the combination of antiangiogenic therapy and immunotherapy has also achieved good results in the treatment of hepatocellular carcinoma. Updated data of IMbrave150 demonstrated that compared with sorafenib in patients with unresectable hepatocellular carcinoma, atezolizumab plus bevacizumab led to significantly improved overall survival (OS:19.2 months vs. 13.4 months, HR = 0.66,95% CI: 0.52–0.85; *p* < 0.001) and progression-free survival (PFS:6.9 months vs. 4.3 months, HR = 0.65,95% CI: 0.53~0.81, *P* < 0.001), and still maintained clinically meaningful survival benefits [72]. Similar to the IMpower150 study, the results of the IMbrave150 study again suggested the possible mechanism of the combination of antivascular therapy and immunotherapy in patients with advanced NSCLC liver metastases from the perspective of tumour microenvironment regulation.

In a recent network meta-analysis of Nine Randomised Controlled Trials (including 1141 patients of NSCLC with liver metastasis), researchers compared the efficacy of different ICIs treatment strategies. Atezolizumab + bevacizumab + chemotherapy and pembrolizumab + chemotherapy showed the greatest benefit for OS and PFS in patients with NSCLC liver metastases. These findings will help clinicians better select treatment strategies for patients with NSCLC liver metastases [73].

In summary, these results suggest that ICIs-based therapy has great potential in the treatment of lung cancer LMs, and is expected to become a systemic treatment for controlling LMs lesions in addition to chemotherapy and targeted therapy. ICIs combination therapy strategy may be superior to ICIs monotherapy, especially immunotherapy combined with antiangiogenic therapy or chemotherapy can bring better curative effect to patients. Taken together, the results of these studies constitute preliminary evidence that ICIs combination therapy strategy does not increase additional risk and strongly supports a role in patients with LMs. Therefore, in this era of immunotherapy, it is of great significance to explore the efficacy and safety of immunotherapy in patients with LMs. We still look forward to more large prospective clinical trials that will provide a higher level of clinical evidence for the best treatment for lung cancer LMs patients.

## ICI combined with local treatment

### ICIs combined with Radiotherapy

The combination of radiotherapy (RT) and immunotherapy for anti-tumour is one of the hot topics in current research. Firstly, there is a strong biological rationale behind combining RT with immunotherapy, with evidence of synergistic effects. The principle of combined RT and immunotherapy was originally derived from the observation of “ abscopal effect”. Similar to chemotherapy, the main goal of RT is to kill tumour cells directly, but in doing so, tumour-specific antigens are released and leads to the “in situ” vaccination effect, thus promote a systemic immune response [74, 75]. In other words, local RT can activate the immune system and induce immune cells to attack tumour cells outside the radiotherapy area, a phenomenon known as the “abscopal effect”. Within the context of immunotherapy, RT has garnered special attention with respect to the so-called “abscopal effect”, whereby RT to one site of disease may induce a broader systemic anti-cancer response. The “abscopal effect” of RT alone is rare, but it can be enhanced in combination with immunotherapy [76].

Preclinical studies have shown that radiotherapy, when combined with immunotherapy, has a positive effect in overcoming the immunosuppressive tumour microenvironment and has a synergistic effect with immunotherapy [77–79]. The combined effects of radiation, anti-CTLA4, and anti-PD-L1 promote response and immunity through different mechanisms [77]. RT can alter the vascular endothelium of the tumour bed and release specific chemokines, thus promoting the recruitment and entry of activated immune cells into the tumour. Modified CD8+ T cells invade the tumour microenvironment and cause local anti-tumour immune response [78]. RT can increase T-cell priming and tumour-specific antigen presentation [79], while enhancing the diversity of the T cell receptor (TCR) repertoire of intratumoral T cells [77]. In addition, repeated irradiation can lead to recruitment and activation of DC. In the case of immune checkpoint blockade, this effect is essential for initiating a systemic anti-tumour response mediated by CD8+ T cells (abscopal effect) [80]. On the other hand, Immunotherapy can enhance the effects of RT. Preclinical studies of RT combined with ICIs in the treatment of NSCLC have shown that RT can increase the expression of PD-L1 in tumour lesions and have an inhibitory effect on tumour immunity, while ICIs can improve this situation [81, 82]. Deng et al. demonstrated that RT-induced tumour regression and synergistically acted with anti-PD-L1 therapy to change the microenvironment of tumour immunosuppression by reducing the local accumulation of tumour-infiltrating myeloid suppressor cells (MDSCs) [81]. Recent studies have shown that immunotherapy can regulate the tumour microenvironment and normalise tumour blood vessels, thereby reducing tumour hypoxia and improving the effect of RT [83, 84].

Based on the results of these preclinical studies, as well as the recognition of improved survival in patients with NSCLC who received pembrolizumab after previously radiotherapy, there is great interest in combining RT with immunotherapy strategies [85]. Clinically, RT is one of the few treatment options for patients with unresectable LMs. Due to the differences in immune microenvironment at different sites, RT at different sites will also have different effects. Studies have found that the immune microenvironment of the liver is highly inhibited, and the expression ratio of inducible T cell co-stimulatory (ICOS) and glucocorticoid-induced tumour necrosis factor receptor (GITR) in tumour tissue of patients receiving local liver RT is higher, and the expression level of PD-1/PD-L1 on tumour cell surface is also higher. This study suggests that RT combined with immunisation may be an effective treatment for patients with NSCLC liver metastases [86]. YU’s team found that the combination of immunotherapy and radiation therapy targeting LMs in a mouse model reduced immunosuppressive macrophages, reduced anti-tumour T cell apoptosis, and restored the antitumor effect of immunotherapy [39]. This exciting result suggests that RT targeting liver metastasis can reverse liver immune tolerance. This combination therapy may actually have a positive impact on the prognosis of patients with LMs [39].

The KEYNOTE-001 study [87] showed that compared with patients who did not receive radiotherapy, patients with metastatic NSCLC who received RT before immunotherapy had better PFS [4.4 months vs. 2.1 months; HR 0.56 (95% CI 0.34–0.91), *p* = 0.019] and OS [10.7 months vs. 5.3 months; HR 0.58 (95% CI 0.36–0.94), *p* = 0.026;]. The synergistic effect of nivolumab with RT was also demonstrated clinically in a retrospective study. Patients who had previously received RT had a significantly higher ORR (36.4%) than those who had not received RT (19%), suggesting that prior RT therapy can improve immunotherapy outcomes and prognosis in patients with advanced NSCLC [88].

Although the combined application of RT and ICIs has strong biological principles support and promising clinical efficacy, there is little evidence on the efficacy and toxicity of RT combined with ICIs in patients with NSCLC liver metastasis. Most scholars believe that RT combined with ICIs is beneficial to the survival of patients with lung cancer liver metastasis, and the combination of the two has been proved safe and effective by many retrospective analyses. RT has an immune-stimulating effect and the effect is rapid, while immunotherapy has a long-term effect, With the emergence of new models of radiation therapy, it is desirable to compare the immune regulation of different models of radiation therapy combined with immunotherapy in this population, but it still needs to be verified by prospective clinical trials.

### ICIs combined with radiofrequency ablation

In addition to RT, radiofrequency ablation (RFA) is also a widely used treatment for intrahepatic metastasis. As the understanding and application of molecular imaging has improved, the application of molecular imaging in the field of interventional therapy has expanded to include minimally invasive treatments guided by cancer images [89]. These locoregional therapies are also used worldwide in the management of primary liver tumours and LMs, and include local tumour ablation [89]. Percutaneous radiofrequency ablation (RFA)is a minimally invasive approach with a favourable safety profile and a lower rate of major complications. Radiofrequency ablation is clinically recommended for patients with oligo metastases (single lesions less than 5 cm or up to 3 lesions less than 3 cm) in the liver [90]. Jackson et al. attempted to compare the efficacy of two modes of treatment, RFA and SBRT. They performed RFA (*n* = 112) or SBRT (*n* = 170) therapy in 161 patients with pathologically diagnosed unresectable LMs. Both treatments were well tolerated and showed good and similar local control for intrahepatic metastases that smaller 2 cm in size. However, for tumours larger than 2 cm, SBRT may be more effective in local control [91].

Some study provides a strong rationale for combining RFA and the PD-L1/PD-1 blockade in the clinical setting [92–94]. Shi et al. found that RFA treatment of liver metastasis simultaneously increased T-cell infiltration and PD-L1 expression in colorectal cancer tissues. They demonstrated that RFA therapy enhanced T-cell-mediated immune response in a mouse tumour model. They established that the combined therapy of RFA and anti-PD-1 antibodies significantly enhanced T-cell immune responses, resulting in stronger antitumor immunity and prolonged survival [92]. RFA can induce the rapid release of large amounts of tumour antigens, to induce tumour-specific T cell response [93], and combination RFA and immune checkpoint inhibitor therapies for LMs might synergistically enhance antitumor immunity. In addition to local intervention, which can increase the activation of tumour-specific T cells, and combined with anti-CTLA4 therapy, which can improve the efficacy of systemic therapy, systemic therapy will be an effective supplement to local therapy, so that residual lesions after ablation can be continuously controlled [94]. Veltri suggest that future researches will require to focus on the beneficial treatments for patients such as particular interventions or combined treatments and also to discover the correct timing of sequential treatments [94].

Considering that there is a strong biological basis for the use of immunotherapy exists in the minimal residual disease state, the efficacy and safety of pembrolizumab after locally ablative therapy (LAT) in oligo-metastatic NSCLC was evaluated in a Phase II study. This study confirmed that patients with low-metastatic NSCLC may benefit from local ablative therapy (LAT), such as surgery or stereotactic radiotherapy. Pembrolizumab in oligometastatic NSCLC after LAT improves PFS without a reduction in quality of life [95].

However, the clinical efficacy of ICIs combined with radiofrequency ablation has been controversial so far, because its effect on survival benefit has not been compared with that of standard treatment. In order to integrate the local interventions within the systemic clinical scenario, it requires more effort to better understand the molecular mechanisms behind residual disease and immune system stimulation of ablation treatments. One of the major challenges for future physicians will be to fully understand the potential synergies between local treatments such as radiofrequency ablation and systemic therapies and translate them into clinical practice.

The above studies showed that ICIs combined with radiotherapy, radiofrequency ablation (RFA)and other local interventions induced the synergistic enhancement of anti-cancer immune response, indicating a great prospect for future cancer treatment.

## predictive markers of LMs

Clinically, the search for predictive biomarkers is critical to identify which populations are likely to obtain clinical benefits from ICIs. To date, several predictors of response to ICIs have been identified, such as PD-L1 expression, tumour mutation burden (TMB) and T-cell infiltration. Multiple studies have shown that PD-L1 expression, TILs, and TMB are biomarkers for predicting ICIs response in NSCLC [96–101]. Many studies have shown that PD-L1 expression is related to the good response to ICIs in NSCLC patients, and support PD-L1 expression as a clinically effective prognostic marker for ICIs [96–99]. The increased expression of PD-L1 can improve the response rate of tumour tissues to ICIs when ICIs is used alone [96, 97].

Several studies have supported the expression of PD-L1 as a predictor of ICIs response in patients with lung cancer liver metastasis. It was found in the liver metastasis subgroup of CheckMate227 that patients with different expression of PD-L1(cutoff 1%) had significant differences in their responses to immunotherapy [55]. Xie et al. reported that immunotherapy significantly prolonged PFS in patients with LMs with positive PD-L1 expression [20]. In a retrospective analysis of Study 1108 and ATLANTIC [44], researchers found that durvalumab had better efficacy in LMs with high PD-L1 expression. Further studies found that PD-L1 ≥ 25% expression in TCs was associated with better OS and ORR and not with liver metastasis, suggesting that PD-L1 expression was an independent factor in predicting the benefit of durvalumab.

It has been suggested that differences in PD‐L1 expression between the metastatic site and the primary tumour may lead to differences in the efficacy of ICIs. For example, PD‐L1 expression is higher in LMs than that in primary neoplasm in advanced colorectal cancer [102]. Similar to metastases from other sites, spatial and temporal heterogeneity in tumour immune characteristics often exists between NSCLC liver metastases and their primary sites [103, 104]. A study of 398 metastatic NSCLC treated with ICIs showed that PD-L1 expression varies substantially across different anatomic sites, being relatively higher in liver than other organs. Studies have found that the predictive value of PD-L1 expression at different biopsy sites for the benefit of ICIs in NSCLC may vary, in which higher PD-L1 expression in distant metastases is significantly associated with better RR, PFS and OS [103]. Researchers have obtained 64 pairs of surgically removed primary tumours and metastatic tumours specimens from 28 NSCLC patients and found that metastatic tumours specimens exhibited higher PD-L1 expression and lower CD8+ cytotoxic T lymphocyte (CTLs) density. Subgroup analyses related to clinical factors showed that the heterogeneity of immune markers was more pronounced in extrapulmonary, metachronous and treated metastatic specimens [104].

In addition to significant heterogeneity in selected biopsied tumour tissues, PD-L1 levels can vary considerably over the course of treatment. PD-L1 expression may change in patients with NSCLC who received anti-cancer therapy during the clinical course. For example, the expression of PD-L1 may be increased in NSCLC patients with EGFR mutations after EGFR-TKI treatment [105, 106]. Omori et al. found that 38% of NSCLC patients had significant changes in PD-L1 expression after receiving anti-cancer treatment [105].

These studies suggest that to evaluating immune markers in these patients with metastatic NSCLC may require multi-focal biopsies as well as repetitive biopsies. Of course, until today, there are still different methods for the determination of PD-L1 in clinical practice and the cut-off level of PD-L1 expression is not fixed [107]. To sum up, PD-L1 expression was only a modest predictor of ICIs response.

More and more studies have also confirmed that tumour mutation burden (TMB) is considered as a surrogate indicator to predict the efficacy of pan-tumour immunotherapy, and is also one of the biomarkers to predict the immune efficacy of metastatic lung cancer in addition to PD-L1 expression [100, 101]. However, there have been no studies using TMB to directly predict liver metastasis of lung cancer.

CD8+T cells are often considered the important effector cell population for ICIs treatment [99]. Tumehet et al. found that CD8 + T cell count at the edge of invasive tumours was significantly reduced in patients with NSCLC liver metastasis [15]. Xie et al. [20] also found that CD8+T cell infiltration in liver lesions was significantly less than that in non-liver lesions. These findings suggest that the poor efficacy of PD-1/PD-L1 inhibitors in the treatment of LMs may be related to the lack of CD8+ T cells. At the same time, Xie et al. [20] also found in the study that the PD-L1 and CD8 double-positive group had a better response to ICIs. These results suggest that the combination of PD-L1 expression and CD8+ T cell infiltration can better predict ICIs response in patients with NSCLC liver metastasis.

Some studies have reported the correlation between driving mutant subtypes and ICIs efficacy, suggesting that KRAS-driven and BRAF-driven subgroups benefit more from ICIs, while EGFR-driven or AlK-driven subgroups benefit more from targeted therapy [108–110]. It is worth noting that about 30% of Kras-mutated NSCLC exhibit lower PD-L1 expression and less tumour-killing immune cell invasion due to SKT11/LKB11 co-mutation, which may lead to poor ICIs efficacy [111, 112].

In summary, preliminary studies suggest that some biomarkers may be associated with immunotherapy response in patients with lung cancer liver metastasis. Due to the heterogeneity of primary and metastatic tumours, the use of biomarker expression in primary lesions to predict survival in patients with LMs is limited. In addition, current immunotherapy prediction models still focus on a single biomarker. Due to the complex immune microenvironment of liver metastasis itself, the immunotherapy of lung cancer LMs becomes challenging. In order to achieve precision cancer therapy, more reliable biomarkers from primary and metastatic sites are needed to predict the immunotherapy effect of lung cancer with liver metastasis, and the integration of clinical variables needs to be actively explored to improve the immunotherapy effect of liver metastasis.

## Summary and Outlook

Immunotherapy has brought the hope of long-term survival for patients with advanced lung cancer, but current studies have shown that immunotherapy especially monotherapy for patients with NSCLC liver metastasis is not effective. Liver metastasis is considered as a predictor of poor prognosis in advanced lung cancer patients treated by ICIs, and how to treat liver metastasis remains an unmet clinical demand.

Antitumor immunity is a complex process, and multiple mechanisms have been proposed to explain liver-induced systemic immune tolerance. From the perspective of mechanism, considering the possible synergistic effect of the combined use of different treatment regimens, some clinical studies related to immunotherapy combined with chemotherapy, antivascular drugs, and RT have shown that it can help improve the prognosis of patients with liver metastasis. Preliminary results from these studies confirmed that the ICIs-based combination therapy has great potential for the treatment of lung cancer LMs, although these patients may achieve less PFS than patients without LMs. However, most of these studies are retrospective analyses (subgroup analysis and observational studies), which should be interpreted with caution. There is no consensus on the safety and efficacy of ICIs and combination therapy in the treatment of lung cancer LMs.

Through our study, we believe that for patients with NSCLC liver metastasis, it is necessary to further explore the characteristics of the liver immune microenvironment in the future, and promote further basic and translational research, which will help to explore potential new therapeutic means, induce the activation of immune cells in the liver, exert anti-tumour function, and thus help to improve the immune tolerance of the liver to the microenvironment. This results in a survival benefit for patients with liver metastasis from NSCLC. At present, new ICIs and personalised vaccines and other therapies have entered the clinical research stage. It is believed that there will be more therapeutic combinations targeting the liver metastasis immune microenvironment in the future. Given that there are few clinical studies focusing on lung cancer patients with liver metastasis at this stage, more clinical and translational studies, especially prospective clinical trials, should be conducted in the future to optimise immunotherapy strategies through systematic evaluation protocols to provide a higher level of clinical evidence for the best treatment of lung cancer patients with LMs.
